# Trends in Breast Cancer Incidence and Stage Distribution Before and During the Introduction of the Mammography Screening Program in Lithuania

**DOI:** 10.1177/1073274818821096

**Published:** 2019-01-06

**Authors:** Laura Steponaviciene, Ruta Briediene, Rasa Vanseviciute, Giedre Smailyte

**Affiliations:** 1 Laboratory of cancer epidemiology, National Cancer Institute, Vilnius, Lithuania; 2 Department of Public Health, Institute of Health Sciences of the Faculty of Medicine of Vilnius University, Vilnius, Lithuania; 3 Department of Radiology, National Cancer Institute, Vilnius, Lithuania; 4 Department of Radiology, Medical Physics and Nuclear Medicine, Vilnius University, Vilnius, Lithuania; 5 Department of Consulting Clinic, National Cancer Institute, Vilnius, Lithuania

**Keywords:** breast cancer, cancer screening, incidence, stage, mammography screening program

## Abstract

**Background:**

The aim of this study was to analyze the incidence trends of localized and advanced breast cancer (BC) before and during the implementation of the mammography screening program (MSP) in Lithuania.

**Methods:**

The study period was divided into 2 intervals: the prescreening period (1998-2005) and implementation period (2006-2012). Analysis was performed for 3 age-groups: 0 to 49 years, 50 to 69 (target population), and older than 70.

**Results:**

In all age-groups, the incidence of localized BC has shown a steady increase, while the incidence of advanced stage BC has decreased. In the target population, during the study period, the stage I BC incidence increased statistically significantly by 10.3% per year (from 3.3 per 100 000 in 1998 to 12.2 per 100 000 in 2012). The increase in localized BC was faster in the period before the implementation of the MSP than during the implementation in 2006 to 2012 (10.3% and 5.7%). A slightly statistically significant decrease was observed for advanced BC during the study period (−1.1% per year), while during the implementation of the MSP, significant changes were not seen.

**Conclusions:**

The results of our study indicate that the implementation of the MSP in Lithuania did not significantly influence trends of localized and advanced BC. Changes observed during the study period, including the prescreening and screening introduction periods, may reflect the general trends in the awareness of BC and improvements in diagnostics.

## Introduction

In Europe, breast cancer (BC) is the most frequently diagnosed neoplasm in women and the leading cancer site of deaths from cancer in women.^1^ In most Western countries, BC mortality has been decreasing since the early 1990s, which is probably due to the combined effect of early detection (partly due to screening and partly due to increasing awareness) and improved treatment.^2-4^

Breast cancer screening is intended to advance the time of diagnosis and thereby improve prognosis. The results of randomized clinical trials conducted in the 1980s in Europe and North America indicate that mammography screening programs (MSPs) reduce the mortality from BC for women aged 50 to 69 years by 20% to 35%.^5^ The stage of BC at the time of diagnosis is associated with the survival rate.^6,7^ If a screening program is effective, the incidence of advanced cancer is expected to decrease, while the incidence of early-stage BC is expected to increase.^8^ It is important to monitor the performance of the national BC screening program from its inception to determine how closely the benefits it achieve approach the benefits seen in the randomized trials and population demonstration projects.^9^

In 2005, the MSP started in Lithuania. In a recent report on Cancer screening in the EU, Lithuania was the country with the lowest participation rate (44.9% in 2014) and 1 of 3 countries where central invitation through a screening registry was not implemented.^10^ The aim of this study was to analyze the incidence trends of localized and advanced BC before and during the implementation of the MSP in the population of Lithuania.

## Materials and Methods

### Data Sources

Analysis was based on data from the population-based Cancer Registry. The Lithuanian Cancer Registry is a population-based cancer registry that contains personal and demographic information (place of residence, sex, date of birth, and vital status) as well as information on the diagnosis (cancer site, date of diagnosis, and method of cancer verification) and death (date of death and cause of death) of all patients with cancer in Lithuania, where the population size is approximately 3 million residents according to the 2011 census.^11^ The principal sources of information on cancer cases are primary, secondary, and tertiary health-care institutions in the country that are responsible for providing notification when cancer is diagnosed. All physicians, all hospitals, and other institutions in the country must send a notification to the Lithuanian Cancer Registry of all cancer cases that come to their attention. Some pathological laboratories send the respective laboratory notification automatically extracted from laboratory data systems, using a standard format. The notifications, which are supplemented by death certificate information, are built into a database suitable for statistical use. This database contains information on all cancer cases diagnosed in Lithuanian residents since 1978. Since the period 1988 to 1992, the Registry data have been included in the “Cancer Incidence in Five Continents.”^12^

The study included all cases of invasive female BC reported to the Registry during 1998 to 2012 (*International Classification of Disease for Oncology, second and third edition, site codes C500-C509, behaviour code 3*). Only invasive BC was included because data on *carcinoma* in situ are not collected by the Registry systematically. During the study period, the TNM system was used for coding the stage of the disease (fourth to seventh editions for periods 1998-2000, 2001-2007, 2008-2010, and 2011-2012, respectively). Corresponding population data by age, sex, and year were available from the Department of Statistics, Lithuania.

### Breast Cancer Screening Program

The Lithuanian BC screening program started in 2005, when the order of the Lithuanian Health Ministry was issued. According to the program, the target population is defined as women aged 50 to 69 years. Women are referred to screening mammography by their general practitioners or gynecologists every 2 years. Mammograms are obtained in 2 views (craniocaudal and mediolateral oblique), which are independently read by 2 radiologists. Both screen-film and digital mammography systems are present. For reporting screening and additional imaging results, the Breast Imaging Reporting and Data System is used, and for the evaluation of breast density, typology according to the American College of Radiology is included.^13^ The assessment information is sent within 2 weeks to the general practitioners.

### Statistical Analysis

The study period was divided into 2 intervals: the prescreening period (1998-2005) and implementation period (2006-2012). Stage I was defined as tumors with T-stage T1 (≤20 mm diameter and no involvement of lymph nodes); advanced stage (stage II+) included tumors T2, T3, and T4 or any number of affected lymph nodes. Analysis was performed for 3 age-groups: 0 to 49 years, 50 to 69 years (target population), and older than 70 years.

Joinpoint regression analysis was used to identify points where a statistically significant change over time in the linear slope of the trend occurred. The annual percentage change (APC) was calculated for the trends by means of the generalized linear model using Joinpoint Software, version 3.4.3.^14^ A maximum number of 3 joinpoints was allowed for the estimations. The APCs were considered statistically significant if *P* < .05. Joinpoint analysis was performed for all ages combined and age- and stage-specific rates.

## Results

From 1998 to 2012, overall 13 874 cases of invasive BC were detected in Lithuanian women. The baseline characteristics of these women are listed in Table 1. A higher proportion of localized BC was detected during the introduction of the MSP period than before (18.9% vs 29.1%), while the proportion of advanced cancer decreased (78.4% vs 63.7%).

**Table 1.** Baseline Characteristics of the Study Group by Period, Before (1998-2005) and During Implementation (2006-2012) of the Mammography Screening Program.

Age-Group	1998-2005		2006-2012	
	N	%	N	%
Total	10 252	100	10 492	100
Age-group				
<50	2551	24.9	2214	21.1
50-69	4828	47.1	5239	49.9
≥7	2873	28.0	3039	29.0
TNM stage				
Stage I	1915	18.7	3054	29.1
Stage II+	8033	78.4	6687	63.7
Unspecified	304	3.0	751	7.2

**Figure 1.**
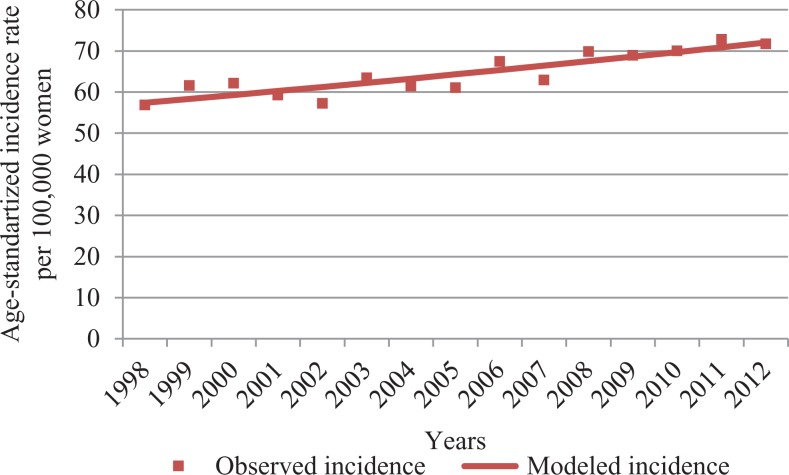
Age-standardized incidence of invasive BC from 1998 to 2012 in Lithuania. BC indicates breast cancer.

**Figure 2.**
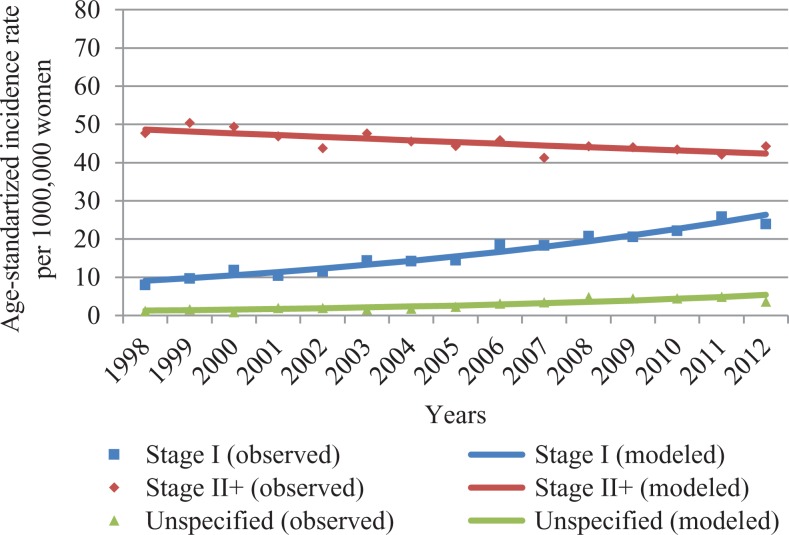
Age-standardized incidence rates by stage (all ages) from 1998 to 2012 in Lithuania.

**Figure 3.**
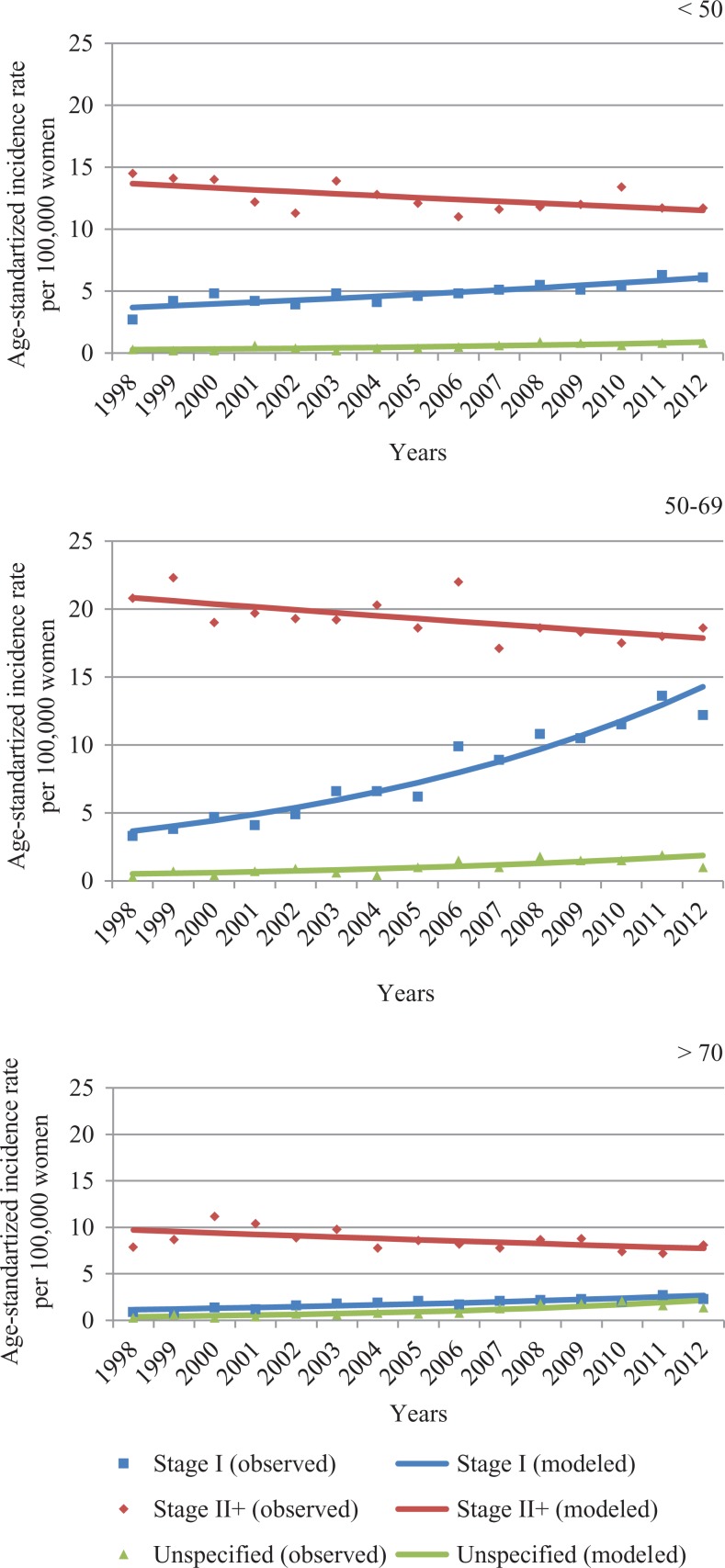
Age-standardized incidence of localized (stage I) and advanced (stage II+) invasive BC by age from 1998 to 2012 in Lithuania. BC indicates breast cancer.

From 1998 to 2012, the age-standardized incidence statistically significantly increased from 56.8 to 71.7 per 100 000 women. The BC incidence rose by 1.6% per year (95% confidence interval: 1.1-2.1). Age-standardized incidence rates can be found in Figure 1. Trends for the incidence of localized and advanced invasive BC by age are presented in Figures 2 and 3.

In all age-groups, the incidence of localized BC showed a steady growth, while the incidence of advanced BC declined. Statistically significant increases in the incidence of localized BC were observed from 1998 to 2012 in all ages combined (+7.9%), in the age group younger than 50 (+3.2%) years, in the target population (+10.2%), and in those older than 70 years (+6.2%). The incidence of the advanced stage statistically significantly decreased by 1.1%, 1.2%, 1.1%, and 1.6% in the all ages, younger than 50 years, 50 to 69 years, older than 70 years groups, respectively.

Time trends for the incidence rates by stage and by period of MSP implementation are shown in Table 2.

**Table 2.** Time Trends of Age-Specific Incidence Rates of BC Before (1998-2005) and During Implementation (2006-2012) of the Mammography Screening Program by Age and Stage in Lithuania.

Age-Group	1998-2005		2006-2012	
	APC	95% CI	APC	95% CI
Total	0.6	−0.9 to 2.1	1.7	0.1 to 3.3
Stage I	8.1	4.4 to 11.9	5.6	2.8 to 8.5
Stage II+	−1.4	−2.8 to 0.0	−0.3	−2.1 to 1.5
Unspecified	7.6	−0.9 to 16.8	3.6	−5.0 to 13.0
<50	−0.7	−3.2 to 1.8	2.2	0.4 to 4.0
TNM stage				
Stage I	3.2	−2.8 to 9.6	4.1	1.5 to 6.9
Stage II+	−2.2	−4.9 to 0.6	1.2	−1.8 to 4.3
Unspecified	4.6	−11.3 to 23.3	4.8	−5.1 to 15.6
50-69	0.9	−0.8 to 2.7	0.8	−2.8 to 4.6
TNM stage				
Stage I	10.3	5.6 to 15.2	5.7	1.6 to 9.9
Stage II+	−1.5	3.4 to 0.4	−1.9	5.7 to 2.0
Unspecified	8.4	6.0 to 24.9	0.4	12.0 to 14.6
≥70	1.4	3.0 to 6.0	1.0	2.3 to 4.5
TNM stage				
Stage I	13.4	8.2 to 18.9	4.7	0.8 to 10.6
Stage II+	−0.8	6.1 to 4.8	−1.2	4.9 to 2.6
Unspecified	10.6	0.1 to 22.5	6.0	8.4 to 22.6

In the target population during the study period, incidence of stage I BC increased from 3.3 per 100 000 in 1998 to 12.2 per 100 000 in 2012. The increase in the localized BC was faster in the period before implementation of the MSP than during implementation from 2006 to 2012 (10.3% and 5.7%). A slightly statistically significant decrease was observed for advanced BC during all the study periods (−1.0% per year), while statistically significant changes were not seen during implementation of the MSP.

In the age-group older than 70 years, similar changes were identified. The incidence of localized BC increased by 13.4% per year before the introduction of the MSP and statistically insignificantly by 4.7% during its introduction. In contrast, in the age-group younger than 50 years, during the introduction of the MSP, the incidence of stage I BC increased statistically significantly. No statistically significant changes in the incidence of advanced BC in the age-group younger than 50 years and in the group over 70 years were identified during the introduction of the MSP.

## Discussion

It can be expected that after the introduction of an MSP, there is an increase in the incidence rates of early-stage cancers, while the incidence rates of advanced cancers decline and remain persistently lowered.^8^ The detection rates of “small cancers” are often specified as a quality assurance measure.^15^ If the screening program significantly reduces the absolute rates of tumors diagnosed at an advanced stage among women attending the screening, a significant reduction in the number of deaths from BC can be observed.^16,17^ A decrease in advanced stage BC some years after the introduction of the MSP is an early surrogate indicator for a reduction in mortality.^16-19^ Therefore, the calculation of the trends in BC stage distribution is widely accepted as a tool for monitoring the MSP.

In our study, we found a statistically significant increase in localized BC and a decrease in advanced BC during all the observational periods—before and during the introduction of the MSP. Data from other studies, with considerable differences in design, tumor stage grouping, duration of observations, and statistical methods, are controversial. Some of them clearly stated a reduction in the incidence rate of advanced BC, associated with the introduction of organized mammography screening.^20-27^ Other studies have demonstrated either no decrease or only nonsignificant declines.^28-33^

In our study, the incidence rates of stage I BC in the target population increased during the study period. A similar trend was seen in the older age-group, which was partly affected by the MSP. In our study during the introduction of the MSP, we also observed a statistically significantly increased incidence of stage I BC in the age-group younger than 50 years.

A study from Rhode Island during an MSP identified a decrease in the median diameter of the tumor from 2 to 1.5 cm with a significant decrease in the incidence of stage III and IV cancers. There was an increase in the incidence of stage I and II BC for women aged 50 to 64 years and in stage I for women older than 65 years.^25^ Investigators from Italy examined the data from 700 municipalities, with a total population of 692 824 women, aged 55 to 74 years, and concluded that a significant and stable decrease in the incidence of late-stage BC was observed from the third year of screening onward, where the incidence rate ratio varied between 0.81 (in years 3-4 of screening) and 0.71 (in years 7-8)^26^. Simbrich et al in Germany observed a marked and statistically significant decline in the advanced BC rates after implementation of an MSP, mainly in women aged 55 to 69 years but not in the adjacent groups.^27^ This decrease was seen after a step-up introduction period. In this study, additionally, the incidence rates of stage I cancers clearly increased after the introduction of the MSP. The effect of the implementation of the Dutch BC screening program was analyzed, which demonstrated that the overall invasive BC rates grew mainly due to an increase in the T1 tumors, and the lymph node negative T1 tumors (T1N0), particularly in the age category 50 to 69.^21^ In women aged 50 to 69, there was a significant decline in the incidence of large tumors with lymph node or distant metastases (T2+/N+/M1). This reduction in advanced disease preceded the observed significant BC mortality reduction to a comparable extent by approximately 2 years. The analysis from the small Dutch region Limburg showed a decrease in the incidence of stage II to IV cancers: The incidence rate of stage II to IV tumors was 10% lower in 1995 (several years after implementation of screening) than the incidence rates from 1987 to 1990 (before screening).^20,24^ A decline by 15% was observed for the incidence rate of node-positive BC 5 years after the MSP was introduced. A study to assess the impact of the National Health Service breast screening program on the overall and stage-specific incidence of BC was conducted in East Anglia.^23^ The study results clearly showed a significant growth in the detection of early-stage cancer in the age-group 50 to 64 years, corresponding with the introduction of screening in the region. The highest incidence increase occurred in 1991, two years after the introduction of screening. The estimated reduction in the advanced stages was 7% to 19% six years after the start of the screening.

In our study, during implementation of the MSP, no statistically significant changes in the incidence of advanced BC were detected in the target population. This corresponds to the data of other studies, where a decrease in the incidence rate of advanced BC was not determined.^28-35^

Having reviewed the Norwegian MSP, Lousdal et al found no decline in the incidence of late-stage cancers in the target population (women 50 to 69 years old) compared to an unscreened group (women 20-49 years old).^33^ The study showed the increase in the annual incidence of localized BC among women aged 50 to 69 years from 63.9 per 100 000 before the introduction of screening to 141.2 per 100 000 afterward. The change in incidence of the localized stage was significantly higher in the age-group that was eligible for screening compared to the younger age-group, with a relative ratio of 1.97 (1.80; 2.17), or *P* < .001, comparing 50 to 69 versus 20 to 49. Nederend et al conducted a population-based study in the Netherlands from 1997 to 2008 with the main aim of determining trends in the incidence of advanced BC with screening mammography.^29^ In this study, after 12 years of biennial screening, a decline in advanced BC was not observed. Jorgensen et al assessed the association between screening in Denmark and tumor size and concluded that BC screening was not associated with a reduction in the incidence of advanced cancer. ^34^ These authors suggest that one-third of tumors diagnosed during screening represent overdiagnosis. Very similar results and conclusions were made by the researches from Australia who evaluated trends in stage-specific BC incidence in New South Wales.^35^ A systematic review by Autier et al focused on incidence trends in areas where mammography screening is practiced.^31^ They stated that in population studies in Europe, North America, and Australia, only small declines in advanced BC incidence were observed, although BC mortality fell dramatically after 1990 in the areas included in this study.

In our study, we found positive changes in the incidence of BC in Lithuania from 1998 to 2012; namely, the incidence of localized BC increased, while the incidence of advanced BC decreased. Changes were seen in all age-groups, those affected by screening and those not affected, which generally reflects the improving oncology situation in our country. If the MSP works as intended, the decrease (at a lower level) in the incidence of advanced stages would also be seen in the women older than 70 years, since a lot of the cancers found at screening will be the ones which without screening would have been diagnosed after 70 years. However, after the initiation of the MSP, the decrease in advanced BC was not statistically significant in this age-group. The improvement in stage distribution can be attributed to notable changes that occurred in Lithuania during the past 2 decades. Major efforts have been made to increase BC awareness. In addition, more advanced imaging equipment was acquired. Digital mammography, breast magnetic resonance imaging, and X-ray-guided stereotactic biopsy became available in the country. However, our analysis cannot distinguish the effect of screening from the effect of the awareness of BC and better diagnostic possibilities. The low participation rate is the main limitation of the MSP in our country. It started at 18% of the targeted population in the first round (2005-2007) and reached 44.9% in the fifth round. It does not reach the recommended levels in European guidelines, and in a recent report on cancer screening in the EU, Lithuania was the country with the lowest participation rate.^10^

Analysis of the trends in BC stage distribution over time is an attractively simple mean of evaluating population-based service screening. This fact has led to a large number of such studies. Such trend studies have several limitations that can influence the results. At first, background incidence rates could be potentially influenced by the changes in risk factors prevalence in general population (obesity, reproductive factors, HRT, and so on). In addition, results can be affected by the extent of opportunistic screening before MSP and the performance indicators of current MSP. The main limitations of our study include: limited follow-up after the introduction of MSP; low participation rate in the screening; opportunistic screening prior to the introduction of an organized program; and no data about carcinoma in situ. Limited follow-up in our study could have an impact on our results, as during the initial screening a lot of advanced cancers will be found, since there has been no previous screening to detect them. Looking at women aged 50 to 69 years in 2006 to 2012 data includes more initial screenings than subsequent screenings, so on it can be hardly expected to find a significant decrease in advanced cancers. In the other countries where the MSP has been identified as having an impact on the decline in advanced-stage BC, the participation rate in the program was much higher, as well as a longer follow-up period was applied. This study is only the first step in evaluating the MSP in Lithuania and its impact on the incidence trends of localized and advanced BC after the start of the MSP. The changes in BC incidence by stage described in the article can be due to problems with the implementation of the program, meaning the lack of participation rather than the usefulness of the MSP.

## Conclusions

The results of our study indicate that the implementation of the MSP in Lithuania did not influence significantly trends of localized and advanced BC. Changes observed during the study period, including the prescreening and screening introduction periods, may reflect the influence of the general trends in the awareness of BC and improvements in diagnostics.
